# Perioperative Management of the Diabetic Patient Referred to Cardiac Surgery

**DOI:** 10.21470/1678-9741-2018-0147

**Published:** 2018

**Authors:** Camila Perez de Souza Arthur, Omar Asdrúbal Vilca Mejía, Gisele Aparecida Lapenna, Carlos Manuel de Almeida Brandão, Luiz Augusto Ferreira Lisboa, Ricardo Ribeiro Dias, Luís Alberto Oliveira Dallan, Pablo Maria Alberto Pomerantzeff, Fabio B. Jatene

**Affiliations:** 1 Cardiovascular Surgery Division, Instituto do Coração do Hospital das Clínicas da Faculdade de Medicina da Universidade de São Paulo (InCor-HCFMUSP), São Paulo, SP, Brazil.

**Keywords:** Cardiac Surgery, Perioperative Management of the Diabetic Patient, Diabetes Mellitus

## Abstract

Currently there is a progressive increase in the prevalence of diabetes in a referred for cardiovascular surgery. Benefits of glycemic management (< 180 mg/dL) in diabetic patients compared to patients without diabetes in perioperative cardiac surgery. The purpose of this study is to present recommendations based on international evidence and adapted to our clinical practice for the perioperative management of hyperglycemia in adult patients with and without diabetes undergoing cardiovascular surgery. This update is based on the latest current literature derived from articles and guidelines regarding perioperative management of diabetic patients to cardiovascular surgery.

**Table:** 

Abbreviations, acronyms & symbols				
**AACE**	**= American Association of Clinical Endocrinologists**		**HbA1c**	**= Hemoglobin A1c**
**ACCORD**	**= Action to Control Cardiovascular Risk in Diabetes**		**ICU**	**= Intensive care unit**
**ADA**	**= American Diabetes Association**		**ITA**	**= Internal thoracic artery**
**ADVANCE**	**= Action in Diabetes and Vascular Disease: Preterax** **and Diamicron MR Controlled Evaluation**		**NICE-SUGAR**	**= Normoglycemia in Intensive Care Evaluation-** **Survival Using Glucose Algorithm Regulation**
**AIA**	**= Anterior interventricular artery**		**NPH**	**= Neutral protamine Hagedorn**
**BITA**	**= Bilateral internal thoracic artery**		**SBD**	**= Brazilian Society of Diabetes**
**CABG**	**= Coronary artery bypass grafting**		**SITA**	**= Single internal thoracic artery**
**DIGAMI**	**= Diabetes and insulin-glucose infusion in acute myocardial infarction**		**SVG**	**= Saphenous vein grafting**
**EASD**	**= European Association for the Study of Diabetes**		**VADT**	**= Veterans Affair Diabetes Trial**
**ECC**	**= Extracorporeal circulation**			

## INTRODUCTION

Diabetic patients have a higher risk of undergoing any type of surgical intervention compared to the non-diabetic population. Additionally, after the procedure, they also present higher perioperative morbidity and mortality^[1]^. Currently, there is a progressive increase in the prevalence of diabetes in patients referred for cardiovascular surgery. Besides higher perioperative morbidity and mortality, these patients present lower angina-free survival and a significant decrease in long-term survival^[2,3]^. In this scenario, evidence shows that appropriate glycemic management can improve these outcomes.

Insulin deficiency and insulin resistance are known to be aggravated by surgery and anesthesia and may lead to lipolysis and ketogenesis, which may result in metabolic acidosis and subsequent electrolyte changes. Protein catabolism increases due to increased degradation and decreased synthesis. Insulin administration may reverse most of these disorders, so, in type 2 diabetic patients, long-acting sulfonylureas, like chlorpropamide, should be discontinued and replaced with agents of short duration. Metformin should always be discontinued. Type 2 diabetic patients with marked hyperglycemia and on oral treatment should receive insulin prior to surgery. Insulin requirements may range from 0.25-0.40 U per gram of glucose in diabetic patients with normal weight undergoing surgery to 0.4-0.8 U per gram of glucose in diabetic patients with obesity, liver disease, on treatment with steroids, or sepsis, and 0.8-1.2 U per gram of glucose in diabetic patients undergoing surgery with use of extracorporeal circulation (ECC). Therefore, the appropriate dose should be personalized, knowing that the protocol preferred by most authors is based on the variable rate of insulin infusion.

Next, we will present recommendations based on international evidence and adapted to our clinical practice for the perioperative management of hyperglycemia in adult patients with and without diabetes undergoing cardiovascular surgery at the Instituto do Coração da Faculdade de Medicina da Universidade de São Paulo.

## PERIOPERATIVE HYPERGLYCEMIA

The limitation in glycemic management is associated with increased morbidity and mortality.

McAlister et al.^[4]^ found, in a retrospective analysis of 291 patients undergoing coronary artery bypass grafting (CABG), that the mean blood glucose level on the first postoperative day adequately predicted the development of an adverse outcome. Harmful effects with elevated intraoperative blood glucose levels were also reported by Gandhi et al.^[5]^ in a retrospective study of 409 patients undergoing cardiac surgery. In this study, pre- and intraoperative hyperglycemias were independent risk factors for perioperative complications, including death.

A retrospective study of 6280 patients undergoing cardiac procedures found that high blood glucose levels were a predictor of mortality in patients with and without diabetes^[6]^. Fish et al.^[7]^ showed that glycemic levels above 250 mg/dL increased 10 times the number of complications in patients undergoing CABG.

Finally, Anderson et al.^[8]^ showed that, in 1375 patients undergoing CABG, preoperative hyperglycemia increased by twice the mortality rate in one year compared to patients with normal preoperative blood glucose.

Together, these studies strongly suggest that hyperglycemia during the perioperative period of cardiovascular surgery is a predictor of morbidity and mortality, regardless of whether or not the patient is diabetic. Next, we will present recommendations supported by evidence of how the reduction of blood glucose levels decreases the incidence of negative outcomes in this group of patients.

Individualization is the key point in elderly population, per the Brazilian Society of Diabetes (SBD), the American Diabetes Association (ADA), and the European Association for the Study of Diabetes (EASD), the fasting glycemic value is up to 150 mg/dL, the postprandial glycemic value is < 180 mg/dL, and the less stringent hemoglobin A1c (HbA1c) goal is < 8%. According to the critical analysis of four major studies - United Kingdom Prospective Diabetes Study (UKPDS), Action in Diabetes and Vascular Disease: Preterax and Diamicron MR Controlled Evaluation (ADVANCE), Action to Control Cardiovascular Risk in Diabetes (ACCORD), and Veterans Affair Diabetes Trial (VADT) - the attempt to rigorously control the glycemia in elderly patients, especially in those with known atherosclerotic diseases, in addition to not preventing cardiovascular events, may increase mortality (ACCORD), possibly but not necessarily due to hypoglycemia. Therefore, the SBD recommends that an overall assessment should be made of each case in order to flexibilize or consolidate the therapeutic targets, following the same principles described^[9]^.

## IMPACT OF GLYCEMIC MANAGEMENT ON CLINICAL RESULTS DURING CARDIAC SURGERY

Benefits of glycemic management (< 180 mg/dL) in diabetic patients during cardiac surgery:

- It reduces mortality;
- It reduces morbidity;
- It decreases the incidence of wound infection;
- It reduces hospital stay;
- It increases long-term survival.

The impact of glycemic management during heart surgery was reported by Furnary et al.^[10]^ analyzing 3554 patients undergoing CABG between 1987 and 2001. Patients were divided into three groups: from 1987 to 1991, they received insulin subcutaneously once every 4 hours to maintain blood glucose level at 200 mg/dL; from 1991 to 1998, continuous intravenous insulin infusion was used to maintain blood glucose levels between 150 and 200 mg/dL; and from 1998 to 2001, the Portland protocol was instituted, administering continuous insulin drip to maintain blood glucose levels between 100 and 150 mg/dL. Continuous infusions of insulin resulted in lower mean glucose levels than that obtained with intermittent subcutaneous insulin therapy. From 1992 and after the establishment of continuous insulin protocols, perioperative mortality in diabetic patients undergoing CABG was reduced by 50% (4.5% *vs*. 1.9%, *P*=0.0001), which was similar to that of non-diabetic patients undergoing CABG. There was also a significant decrease in the incidence of surgical wound infections (*P*=0.001). Furnary et al.^[11]^ expanded its original series to include an additional population of 1980 patients treated with the Portland protocol between 2001 and 2005. They introduced a new method for assessing glycemic management, called "Glucose 3", which consisted of the mean glucose values obtained in surgery and on the first and second postoperative days. An increase in "Glucose 3" was an independent predictor of perioperative mortality (*P*<0.001). On the other hand, this value was also related to an increase in the incidence of deep infections of the sternum, time of hospitalization, blood transfusions, atrial fibrillation, and low cardiac output syndrome.

The importance of the use of insulin in diabetic patients undergoing CABG was presented by Lazar et al.^[12]^ and consists of a modified glucose, insulin, and potassium solution. In this study, 141 diabetic patients undergoing CABG were randomized to receive glucose, insulin, and potassium to maintain a blood glucose level between 120 and 180 mg/dL or a mobile insulin scale to maintain glucose level < 250 mg/dL. The combination of glucose, insulin, and potassium was initiated in the induction of anesthesia and continued for 12 hours in the intensive care unit (ICU). This protocol obtained better glycemic management immediately before cardiopulmonary bypass (169 mg/dL *vs*. 209 mg/dL, *P*<0.0001) and after 12 hours in the ICU (134 mg/dL *vs*. 266 mg/dL, *P*<0.0001). Patients treated with strict glycemic control had higher cardiac indices (*P*<0.0001) and less need for vasoactive drugs (*P*<0.05) and cardiac stimulation (*P*<0.05). Strict glycemic control resulted in a lower incidence of infections (0 *vs*. 13%, *P*=0.01) and atrial fibrillation (15% *vs*. 60%, *P*=0.007). All this contributed to the reduction of hospitalization time (6.5 days *vs*. 9.2 days, *P*=0.0003). After five years, Kaplan-Meier curves showed survival advantage (*P*=0.04) for patients who received better glycemic control. They had a lower incidence of recurrent angina (*P*=0.01), wound infection (*P*=0.03), and were able to maintain a lower class of angina (*P*=0.03).

Evidence on the importance of rigorous glycemic management in patients undergoing CABG has also been demonstrated in a study by Van den Berghe et al.^[13]^, involving 1548 patients on mechanical ventilation admitted to a surgical ICU. In this prospective, randomized study, 62% of the patients underwent cardiac surgery and only 13% had a history of diabetes. Patients were randomized into a conventional therapeutic group, in which insulin was administered only if the blood glucose level was > 215 mg/dL to maintain a target of 180-200 mg/dL, and into another group that received continuous infusion of insulin to maintain glucose levels between 80 and 110 mg/dL. Intensive insulin therapy resulted in a significant reduction in mortality (10% *vs*. 20%, *P*=0.005). Cardiac surgery mortality was only reduced in those patients who required three days of ICU care. Hospital mortality for all cardiac surgery patients, regardless of ICU stay, was reduced from 5.1% to 2.1% (*P*<0.05). Intensive glycemic management had no effect on the morbidity and mortality of patients who stayed more than three days in the ICU. In another study to identify patients who could benefit more from strict glycemic management, D'Alessandro et al.^[14]^ sought to correlate the effect of strict glycemic management with the results expected by EuroSCORE in diabetic patients undergoing CABG. Using the propensity score, 300 diabetic patients undergoing CABG who received strict glycemic management (150 to 200 mg/dL in the operating room, 140 mg/dL in the ICU) and 300 diabetic patients undergoing CABG when the insulin protocols did not yet exist were compared. The rigid glycemic management group had a significantly lower mortality than that expected by EuroSCORE (1.3% *vs*. 4.3%, *P*=0.01). Mortality was low, mainly in the highest risk group (EuroSCORE 4; 2.5% *vs*. 8.0%, *P*=0.03). In contrast, there was no difference between observed and expected mortalities in the group without strict glycemic management in patients with EuroSCORE < 4. Two other additional studies showed the importance of glycemic management in reducing sternal infection. Zerr et al.^[15]^ studied the effects of glycemic management on the incidence of sternal infection in 1585 diabetic patients undergoing CABG. Sternal infection increased by 1.3% in patients with mean glucose values of 100 to 150 mg/dL to 6.7% in patients with levels of 250 to 300 mg/dL. In a retrospective study of diabetic patients undergoing CABG, Hruska et al.^[16]^ observed that continuous infusion of insulin maintaining blood glucose levels between 120 and 160 mg/dL significantly reduced the incidence of sternal infection compared with intermittent subcutaneous injections.

## GLYCEMIC MANAGEMENT DURING CARDIAC SURGERY IN NON-DIABETIC PATIENTS

Intraoperative glycemic management with continuous infusion of intravenous insulin is not necessary in non-diabetic patients undergoing cardiac surgery, provided that blood glucose levels remain < 180 mg/dL.

Is rigorous glycemic control necessary for all patients undergoing cardiovascular surgery?

Butterworth et al.^[17]^ showed the effects of strict blood glucose control in 381 non-diabetic patients undergoing isolated CABG. In this prospective, randomized study, one group received continuous infusion of insulin when intraoperative blood glucose levels were > 100 mg/dL. The other group did not receive insulin coverage. The primary outcome was the incidence of new neurological events, neuro-ophthalmologic and neurobehavioral deficits, and neurological-related deaths. Intraoperative blood glucose levels were significantly lower in patients receiving insulin infusion, although there was no difference in the incidence of neurological complications. Likewise, there were no differences between the groups regarding operative mortality, need for vasoactive drugs, and time of hospitalization; however, patients who did not receive intraoperative insulin had blood glucose levels at 200 mg/dL. In this study, intraoperative glycemic management did not improve short- and long-term clinical outcomes in the non-diabetic patients' group.

Gandhi et al.^[18]^ studied the effects of intensive intraoperative insulin therapy in 400 patients undergoing elective CABG. Patients were randomized prospectively into a group with continuous insulin, to maintain blood glucose levels between 80 and 100 mg/dL, and into a conventional group, to maintain blood glucose levels up to 200 mg/dL using intermittent boluses of intravenous insulin. The incidence of diabetes was 20% in both groups. There was no difference between the groups in the composite outcome: death, sternal infection, prolonged ventilation, cardiac arrhythmias, stroke, and renal failure up to 30 days after surgery. There was also no difference in ICU time and hospital admission between the groups. And there was a tendency for more deaths (*P*=0.06) and stroke (*P*=0.02) in the intensive care group with insulin. The limitations of this study were the inclusion of patients with and without diabetes and that both groups received intensive insulin therapy in the immediate postoperative period.

## PERIOPERATIVE HYPERGLYCEMIA MANAGEMENT USING INSULIN PROTOCOLS

### Recommendation: Class I

- Glycemic management is best achieved with continuous insulin infusions instead of subcutaneous injections of insulin or intermittent intravenous insulin bolus (level of evidence A).
- All diabetic patients undergoing cardiac surgery should receive continuous insulin infusion during surgery and for at least 24 hours postoperatively to maintain blood glucose levels ≤ 180 mg/dL (level of evidence B).

Intravenous insulin therapy is the preferred method for perioperative insulin administration. It allows rapid titration, facilitating glycemic management during periods of malabsorption and deficiency or insulin resistance^[19]^. Table 1 describes various protocols available for the use of insulin and the glycemic values that can be achieved.

**Table 1:** Types of insulin infusion protocols.

Glycemic control protocol	Brief description	mg/dL
Markovitz	Five algorithms with precalculated rates using the multiplier; infusion rates are determined by blood glucose range	120-199
Leuven	General guidelines on insulin drip titration	80-110
Yale	Calculated rates based on glycemic value and rates of change	90-120
Portland	Specific infusion rates in insulin boluses according to blood glucose level; five categories are available for ICU and infirmary	70-110 80-120 100-150 125-175 150-200
DIGAMI	Specific rates of insulin infusion per blood glucose range	126-180
Washington University	Four algorithms with rates precalculated by the multiplier; infusion rates are determined by blood glucose range	80-180
Atlanta Medical Center	Ten algorithms with rates precalculated by multiplier; infusion rates are determined by blood glucose range	80-110
Glucommander	Infusion rates calculated by computer according to programmed algorithms	80-120
Clarian	Infusion rates calculated by computer according to algorithms programmed by GlucoStabilizer	80-110
Matias	Infusion rates calculated by computer according to based algorithm of absolute glucose value	80-110
eMPC	Infusion rates calculated by computer based on model of predictive control algorithm with variable sampling rate	80-110

DIGAMI=diabetes and insulin-glucose infusion in acute myocardial infarction; ICU=intensive care unit

Source: Friedberg et al.^[19]^, 2006 and update^[^20^,^21^]^.

The choice of a specific protocol depends on the needs and resources of the institution. Thus, to ensure the effective and safe execution of any protocol, individuals involved in patient care should feel comfortable.

The success of any protocol can be determined based on results such as: the time taken to reach the target value, the specific concentrations of glycemic handling, the mean blood glucose level, the percentage of blood glucose levels desired, or the calculation of an area under the curve of the percentage of time taken to reach a certain interval^[22]^. This issue is specifically addressed by the American Association of Clinical Endocrinologists (AACE) on the management of hyperglycemia in the hospital^[23,24]^. Also, for patient's follow-up and safety, the number (or percentage) of hypoglycemic events and any other intercurrence should be recorded.

## PREOPERATIVE MANAGEMENT AND EVALUATION FOR DIABETIC PATIENTS

### Recommendations:

#### Class I

- Diabetic patients receiving continuous insulin (lispro, aspart, glulisine, or regular) should maintain it until after dinner in the night before surgery (level of evidence B).
- The schedule of insulin therapy to achieve glycemic management should be initiated using a combination of short- and long-acting subcutaneous insulin or an insulin infusion protocol in hospitalized patients awaiting surgery (level of evidence C).
- All hypoglycemic agents and non-insulin oral diabetes medications should be maintained up to 24 hours before surgery (level of evidence C).
- The level of glycated HbA1c should be assessed prior to surgery in patients with diabetes or those at risk for postoperative hyperglycemia to characterize the level of preoperative glycemic management (level of evidence C).

#### Class II

- Before surgery, it is reasonable to maintain blood glucose level ≤ 180 mg/dL (level of evidence B).

Efforts should be made to optimize glycemic control prior to surgery and to avoid an increase in morbidity, including a higher incidence of surgical wound infections and an increase in the postoperative period^[11,12]^. In general, all oral antidiabetic agents should be discontinued 24 hours before surgery, especially sulfonylureas (*e.g*., glipizide) and glinides (*e.g*., nateglinide or repaglinide). These drugs can induce hypoglycemia in the absence of food. Patients who take insulin and are admitted on the day of surgery should be instructed to continue basal insulin use (*e.g*., glargine, detemir, or neutral protamine Hagedorn [NPH]) and maintain insulin by nutritional need (*e.g*., lispro, aspart, glulisine, or regular), unless it is contraindicated by the responsible physician. Before surgery, NPH insulin can be reduced by half or one third to prevent hypoglycemia. To achieve rapid control in the hospitalized patient with hyperglycemia (persistent glycemia > 180 mg/dL for a period > 12 hours prior to surgery), insulin therapy should be used either by continuous rapid intravenous or subcutaneous infusion.

In hyperglycemic patients, on the day of surgery, intravenous insulin therapy is an effective alternative to achieve rapid control. In addition, patients with a known history of diabetes (type 1 or 2) may initiate preoperative intravenous therapy. The patient should be questioned about other medications used preoperatively, because of their potential for insulin resistance. These include steroids, protease inhibitors, and antipsychotic medications. It is also necessary to emphasize the importance of the identification of patients with kidney failure because the decrease of insulin clearance increases the risk of hypoglycemia.

Glycosylated HbA1c is an accurate indicator of glycemic control over a period of 2 to 3 months. ADA reports that adequate glycemic control is associated with an HbA1c ≤ 7%^[25]^. Therefore, preoperative assessment of HbA1c in diabetic patients or at risk of postoperative hyperglycemia helps to optimize the glycemic management of those with elevated levels of HbA1c. Hence, it facilitates the identification of patients who may require more aggressive glycemic management after discharge from hospital.

## INTRAOPERATIVE MANAGEMENT

### Recommendations:

#### Class I

- Blood glucose levels > 180 mg/dL in non-diabetic patients occurring solely during ECC may be initially treated with single or intermittent intravenous insulin dosing as long as blood glucose levels ≤ 180 mg/dL are maintained. However, in patients with persistently elevated blood glucose levels (> 180 mg/dL) after ECC, insulin drip should be instituted and requested to be evaluated by an endocrinologist (level of evidence B).
- If infusion of intravenous insulin is initiated preoperatively, it should be continued throughout intraoperative and immediate postoperative periods according to institutional protocols to maintain blood glucose level ≤ 180 mg/dL (level of evidence C).

Patients receiving infusion of intravenous insulin should have their blood glucose monitored every 30 to 60 minutes. A monitoring every 15 minutes should be performed for periods of rapid sensitivity change, such as during cardioplegia administration and in cooling or systemic heating. Patients who started preoperative intravenous infusion should continue intraoperatively to maintain a blood glucose level ≤ 180 mg/dL.

Patients with no history of diabetes may present transient elevation of blood glucose level (> 180 mg/dL) during ECC. These patients may have insulin resistance and should be treated with a single or intermittent dose of insulin to maintain blood glucose level ≤ 180 mg/dL. We should be cautious in initiating continuous intravenous insulin in these cases, because insulin requirements may decrease rapidly in the immediate postoperative period, resulting in severe hypoglycemia^[25]^. However, non-diabetic patients with persistently elevated blood glucose levels (> 180 mg/dL) during surgery should receive insulin drip. As a large percentage of these patients may develop diabetes, the evaluation of the endocrinologist should be obtained postoperatively.

## STRATEGIES FOR MYOCARDIAL REVASCULARIZATION SURGERY IN DIABETIC PATIENTS

1. The FREEDOM study was designed to evaluate the benefits of current angioplasty techniques (which uses drug-eluting stents) and of CABG in combination with aggressive drug therapy in diabetic patients^[26]^. The results of overall mortality, acute myocardial infarction, and stroke were significantly lower in the surgery group. This study confirmed that CABG is the preferred revascularization strategy in diabetic patients.
2. Since the 1980s, grafting with the internal thoracic artery (ITA) or mammary artery to the anterior interventricular artery (AIA) or anterior descending artery has significantly increased survival and it is associated with a lower incidence of late cardiac events and a better quality of life in a 10-year follow-up^[27]^. This benefit results from a higher long-term patency of ITA when compared to saphenous vein grafts.
3. The use of ITAs (double mammary) is associated with better survival rate compared to the use of a single ITA in both diabetic and non-diabetic patients. However, studies show that the use of ITAs is associated with a higher occurrence of deep sternal infection in relation to the use of a single ITA. This risk of deep sternal infection is counterbalanced by a long-term benefit, as it has recently been reported in the literature that deep sternal infection did not limit the benefit of ITAs in the long-term^[28]^. In addition, the risk of infection can be minimized by modifying the technique of ITAs dissection. In this sense, the skeletonization of ITA (*i.e*., isolated dissection of ITA) is preferable to the removal of the pedicled ITA (*i.e*., including tissue and vein) and thus, collateral branches and vascularization of the sternum can be preserved, with improvement in infection rates of the sternum, particularly in diabetic patients.
4. The ESC/EACTS 2014 guidelines on myocardial revascularization recommend that the use of both ITAs should be considered in patients younger than 70 years of age (Class IIa). The guidelines also establish that, in diabetic patients with multiarterial coronary disease and with acceptable surgical risk, CABG is recommended for percutaneous coronary intervention (Class I) and that the use of both ITAs should be considered in these patients. Thus, all diabetic patients younger than 70 years of age, without morbid obesity, and whose HbA1c dosage is < 7% should receive double mammary graft. On the other hand, skeletonized dissection of ITAs is recommended^[27]^.
5. A sub-analysis of the ROOBY study compared the clinical evolution between CABG with and without the use of ECC in diabetic patients. This analysis showed higher rates of incomplete revascularization that correlated with an increase in the rates of adverse events and 30-day mortality in diabetic patients operated on without ECC. In addition, in this group of patients, graft patency at one year was significantly lower in non-ECC surgeries (83.1%) than in ECC surgeries (88.4%)^[29]^ (Figure 1).
  **Fig. 1:**
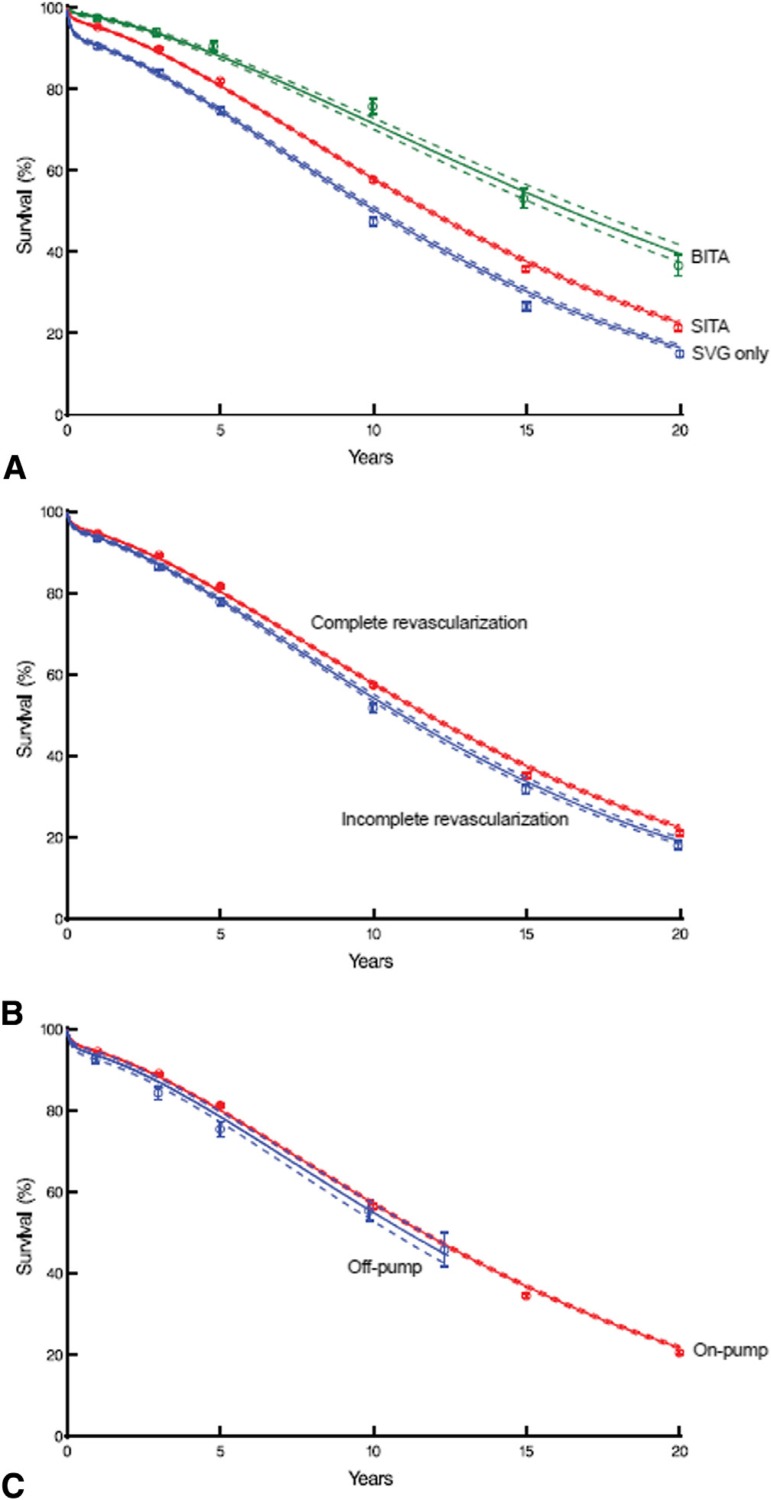
Survival curves after CABG in diabetic patients. Each symbol represents a death at 1, 3, 5, 10, 15, and 20 years after surgery as estimated by Kaplan-Meier. Vertical bars are confidence intervals with standard error of ± 1. Continuous lines are parametric estimates within intermittent lines with standard error ± 1. A: Stratification by both ITAs (BITA and SITA) isolated and only saphenous vein (SVG). B: Stratification according to complete versus incomplete revascularization. C: Stratification according to CABG with ECC (onpump) and CABG without ECC (off-pump) ^[29]^. BITA=bilateral internal thoracic artery; CABG=coronary artery bypass grafting; ECC=extracorporeal circulation; ITA=internal thoracic artery; SITA=single internal thoracic artery; SVG=saphenous vein grafting

## GLYCEMIC MANAGEMENT IN ICU

### Recommendations: Class I

- Patients with or without diabetes and persistently elevated blood glucose levels (> 180 mg/dL) should receive intravenous insulin infusions to maintain blood glucose levels ≤ 180 mg/dL during their stay in ICU (level of evidence A).
- Patients requiring ≥ 3 days in ICU due to ventilator dependence or the need for inotropes, continuous venovenous hemodialysis or hemofiltration, and antiarrhythmic intra-aortic balloon or left ventricular assist device should receive continuous infusion of intravenous insulin to maintain a blood glucose level < 150 mg/dL, regardless of whether or not they are diabetics (level of evidence B).
- Before discontinuing continuous infusion of intravenous insulin, patients should be scheduled for subcutaneous insulin administration using institutional protocols (level of evidence B).

Persistently elevated glucose levels in patients with or without previous diabetes ) should receive intravenous infusion of insulin to maintain blood glucose levels ≤ 180 mg/dL.Those requiring a time greater than or equal to 3 days of ICU, prolonged ventilatory support, inotropic drugs or technical support, renal insufficiency, antiarrhythmic therapy should be infused with continuous insulin to maintain a blood glucose level of <150 mg/dL ^[11-13]^.

Patients receiving continuous infusion of intravenous insulin in ICU should have their blood glucose monitored at least every hour until they are stable. This frequency avoids fluctuations in blood glucose levels and minimizes the risk of hypoglycemia, which fortunately is a rare complication with minimal risk of morbidity^[11-13]^. Once patients are scheduled to be discharged from the ICU, they should switch to a subcutaneous insulin regimen. Daily insulin requirements can be calculated based on the average amount of insulin required in the last 24 hours and the patients' new nutritional regimen^[30]^.

The Normoglycemia in Intensive Care Evaluation-Survival Using Glucose Algorithm Regulation (NICE-SUGAR) compared two glycemic control strategies based on insulin (target blood glucose <180 mg/dL in the control group and a range of 81-108 mg/dL in the intervention group) in a sample of 6104 ICU patients. In this study, the intensive blood glucose control was associated with higher cardiovascular mortality, with an absolute difference of 5.8%. Kansagara et al.^[31]^ reported that intensive postoperative blood glucose control was associated with a reduction in the composite endpoint of all-cause mortality, myocardial infarction, and acute heart failure.

Moderately strict glycemic control added to the usual standard of care in patients undergoing cardiac surgery were associated with a 6% reduction in infection rates and a 12% reduction in atrial fibrillation, with no differences between groups in terms of mortality^[32]^.

## GLYCEMIC MANAGEMENT IN THE WARD

### Recommendations: Class I

- A maximum blood glucose level < 180 mg/dL should be achieved in the postprandial state (level of evidence B).
- A fasting and before meals blood glucose level of ≤ 110 mg/dL should be reached after transfer to the ward (level of evidence C).
- Oral antidiabetics should be restarted in patients with adequate blood glucose levels, with few exceptions. Consequently, insulin doses should be reduced (level of evidence C).
- According to the AACE, a reasonable goal for a noncritical patient in a hospital ward is fasting glucose < 110 mg/dL and postprandial glucose < 180 mg/dL^[27]^. On the other hand, the best method to achieve this control is with programmed subcutaneous insulin (glargine or determir) or bolus therapy (lispro, aspart, or glulisine). Patients with type 2 diabetes who take preoperative oral antidiabetics may restart these medications after reaching adequate blood glucose levels and starting a regular diet. However, metformin should not be restarted until it reaches a stable renal function. Glitazones cannot be restarted in patients with congestive heart failure or hepatic dysfunction.

## PREPARATION FOR HOSPITAL DISCHARGE

### Recommendations: Class I

- Before discharge, all patients with diabetes and those who started a new glycemic management regimen should receive guidance on blood glucose monitoring, drug administration, diet, and lifestyle changes (level of evidence C).
- After discharge, changes in glycemic control therapy should be reported to primary care physicians and an endocrinologist appointment should be scheduled if necessary (level of evidence C).

All patients with hyperglycemia after cardiac surgery should be evaluated by a diabetes team to define glycemic control after discharge. When hyperglycemia is first discovered in the perioperative period, if insulin is administered for the first time, or when a new insulin protocol is instituted, the patient should receive expert advice before discharge. Guidance on glycemic monitoring, drug administration, feeding, exercise, and lifestyle changes should be initiated at least two days before discharge^[33-35]^.

## FUTURE RESEARCH AREAS

Important issues regarding the management of hyperglycemia during cardiovascular surgery should still be elucidated. In this scenario, future research will determine: (1) the ideal level of glycemic management and, if appropriate, the most important perioperative period to maintain this control; (2) whether the blood glucose level reached is as important as the amount of insulin delivered; (3) the importance of preoperative levels of HbA1c and whether surgery should be postponed in patients with high HbA1c levels; (4) if the handling of elderly patients should be protocolized or individualized, could them tolerate higher levels of HbA1c without risk morbidities or their increased risk of hypoglycemia is so higher than of the general population that a more permissive HbA1c would significantly reduce their morbidity and mortality? Answers to these questions will increase our understanding of hyperglycemia during cardiovascular surgery and will help us determine more appropriate methods to achieve glycemic control and improve clinical outcomes in this group of high-risk patients.

**Table:** 

**Authors’ roles & responsibilities**	
CPSA	Substantial contributions to the conception or design of the work; or the acquisition, analysis, or interpretation of data for the work; drafting the work or revising it critically for important intellectual content
OAVM	Substantial contributions to the conception or design of the work; or the acquisition, analysis, or interpretation of data for the work; drafting the work or revising it critically for important intellectual content
GAL	Drafting the work or revising it critically for important intellectual content; final approval of the version to be published
CMAB	Drafting the work or revising it critically for important intellectual content; final approval of the version to be published
LAFL	Drafting the work or revising it critically for important intellectual content; final approval of the version to be published
RRD	Drafting the work or revising it critically for important intellectual content; final approval of the version to be published
LAOD	Drafting the work or revising it critically for important intellectual content; final approval of the version to be published
PMAP	Drafting the work or revising it critically for important intellectual content; final approval of the version to be published
FBJ	Drafting the work or revising it critically for important intellectual content; final approval of the version to be published

## References

[1] Peters A, Kerner W (1995). Perioperative management of the diabetic patient. Exp Clin Endocrinol Diabetes.

[2] Szabó Z, Hakanson E, Svedjeholm R (2002). Early postoperative outcome and medium-term survival in 540
diabetic and 2239 nondiabetic patients undergoing coronary artery bypass
grafting. Ann Thorac Surg.

[3] Carson JL, Scholz PM, Chen AY, Peterson ED, Gold J, Schneider SH (2002). Diabetes mellitus increases short-term mortality and morbidity in
patients undergoing coronary artery bypass graft surgery. J Am Coll Cardiol.

[4] McAlister FA, Man J, Bistritz L, Amad H, Tandon P (2003). Diabetes and coronary artery bypass surgery: an examination of
perioperative glycemic control and outcomes. Diabetes Care.

[5] Gandhi GY, Nuttall GA, Abel MD, Mullany CJ, Schaff HV, Williams BA (2005). Intraoperative hyperglycemia and perioperative outcomes in
cardiac surgery patients. Mayo Clin Proc.

[6] Doenst T, Wijeysundera D, Karkouti K, Zechner C, Maganti M, Rao V (2005). Hyperglycemia during cardiopulmonary bypass is an independent
risk factor for mortality in patients undergoing cardiac
surgery. J Thorac Cardiovasc Surg.

[7] Fish LH, Weaver TW, Moore AL, Steel LG (2003). Value of postoperative blood glucose in predicting complications
and length of stay after coronary artery bypass grafting. Am J Cardiol.

[8] Anderson RE, Brismar K, Barr G, Ivert T (2005). Effects of cardiopulmonary bypass on glucose homeostasis after
coronary artery bypass surgery. Eur J Cardiothorac Surg.

[9] Sociedade Brasileira de Diabetes (2017). Diretrizes da Sociedade Brasileira de Diabetes 2017-2018.

[10] Furnary AP, Gao G, Grunkemeier GL, Wu Y, Zerr KJ, Bookin SO (2003). Continuous insulin infusion reduces mortality in patients with
diabetes undergoing coronary artery bypass grafting. J Thorac Cardiovasc Surg.

[11] Furnary AP, Wu Y, Bookin SO (2004). Effect of hyperglycemia and continuous intravenous insulin
infusions on outcomes of cardiac surgical procedures: the Portland Diabetic
Project. Endocr Pract.

[12] Lazar HL, Chipkin SR, Fitzgerald CA, Bao Y, Cabral H, Apstein CS (2004). Tight glycemic control in diabetic coronary artery bypass graft
patients improves perioperative outcomes and decreases recurrent ischemic
events. Circulation.

[13] van den Berghe G, Wouters P, Weekers F, Verwaest C, Bruyninckx F, Schetz M (2001). Intensive insulin therapy in the critically ill
patients. N Engl J Med.

[14] D'Alessandro C, Leprince P, Golmard JL, Ouattara A, Aubert S, Pavie A (2007). Strict glycemic control reduces EuroSCORE expected mortality in
diabetic patients undergoing myocardial revascularization. J Thorac Cardiovasc Surg.

[15] Zerr KJ, Furnary AP, Grunkemeier GL, Bookin S, Kanhere V, Starr A (1997). Glucose control lowers the risk of wound infection in diabetics
after open heart operations. Ann Thorac Surg.

[16] Hruska LA, Smith JM, Hendy MP, Fritz VL, McAdams S (2005). Continuous insulin infusion reduces infectious complications in
diabetics following coronary surgery. J Card Surg.

[17] Butterworth J, Wagenknecht LE, Legault C, Zaccaro DJ, Kon ND, Hammon Jr JW (2005). Attempted control of hyperglycemia during cardiopulmonary bypass
fails to improve neurologic or neurobehavioral outcomes in patients without
diabetes mellitus undergoing coronary artery bypass grafting. J Thorac Cardiovasc Surg.

[18] Gandhi GY, Nuttall GA, Abel MD, Mullany CJ, Schaff HV, O'Brien PC (2007). Intensive intraoperative insulin therapy versus conventional
glucose management during cardiac surgery: a randomized
trial. Ann Intern Med.

[19] Friedberg SJ, Lam YW, Blum JJ, Gregerman RI (2006). Insulin absorption: a major factor in apparent insulin resistance
and the control of type 2 diabetes mellitus. Metabolism.

[20] Hovorka R, Kremen J, Blaha J, Matias M, Anderlova K, Bosanska L (2007). Blood glucose control by a model predictive control algorithm
with variable sampling rate versus a routine glucose management protocol in
cardiac surgery patients: a randomized controlled trial. J Clin Endocrinol Metab.

[21] Blaha J, Kopecky P, Matias M, Hovorka R, Kunstyr J, Kotulak T (2009). Comparison of three protocols for tight glycemic control in
cardiac surgery patients. Diabetes Care.

[22] Braithwaite SS, Godara H, Song HJ, Rock P (2006). No patient left behind: evaluation and design of intravenous
insulin infusion algorithms. Endocr Pract.

[23] Goldberg PA, Bozzo JE, Thomas PG, Mesmer MM, Sakharova OV, Radford MJ (2006). "Glucometrics": assessing the quality of inpatient glucose
management. Diabetes Technol Ther.

[24] (2018). American Diabetes Association & Pharmacologic approaches to
glycemic treatment: Standards of Medical Care in Diabetes -
2018. Diabetes Care.

[25] Chaney MA, Nikolov MP, Blakeman BP, Bakhos M (1999). Attempting to maintain normoglycemia during cardiopulmonary
bypass with insulin may initiate postoperative hypoglycemia. Anesth Analg.

[26] Farkouh ME, Domanski M, Sleeper LA, Siami FS, Dangas G, Mack M, FREEDOM Trial Investigators (2012). Strategies for multivessel revascularization in patients with
diabetes. N Engl J Med.

[27] Raza S, Sabik 3rd JF, Masabni K, Ainkaran P, Lytle BW, Blackstone EH (2014). Surgical revascularization techniques that minimize surgical risk
and maximize late survival after coronary artery bypass grafting in patients
with diabetes mellitus. J Thorac Cardiovasc Surg.

[28] Jatene FB, Kolh P (2015). Bilateral internal thoracic artery grafts for myocardial
revascularization in insulin dependent diabetic patients: time for wide
clinical practice?. Eur J Cardiothorac Surg.

[29] Shroyer AL, Hattler B, Wagner TH, Baltz JH, Collins JF, Carr BM, VA #517 Randomized On/Off Bypass (ROOBY) Study Group (2014). Comparing off pump and on pump clinical outcomes and costs for
diabetic cardiac surgery patients. Ann Thorac Surg.

[30] Schmeltz LR, DeSantis AJ, Schmidt K, O'Shea-Mahler E, Rhee C, Brandt S (2006). Conversion of intravenous insulin infusions to subcutaneously
administered insulin glargine in patients with hyperglycemia. Endocr Pract.

[31] Kansagara D, Fu R, Freeman M, Wolf F, Helfand M (2011). Intensive insulin therapy in hospitalized patients: a systematic
review. Ann Intern Med.

[32] Viana MV, Moraes RB, Fabbrin AR, Santos MF, Gerchman F (2014). Assessment and treatment of hyperglycemia in critically ill
patients. Rev Bras Ter Intensiva.

[33] Loures DRR, Carvalho RG, Mulinari L, Silva Jr. AZ, Schmidlin CA, Brommelströet M (2000). Cardiac surgery in elderly patients. Rev Bras Cir Cardiovasc.

[34] Ko SH, Song KH, Kim SR, Lee JM, Kim JS, Shin JH (2007). Long-term effects of a structured intensive diabetes education
programme (SIDEP) in patients with type 2 diabetes mellitus: a 4-year
follow-up study. Diabet Med.

[35] Fritsche A, Stumvoll M, Goebbel S, Reinauer KM, Schmülling RM, Häring HU (1999). Long term effect of a structured inpatient diabetes teaching and
treatment programme in type 2 diabetic patients: influence of mode of
follow-up. Diabetes Res Clin Pract.

